# Focus stacking single-event particle radiography for high spatial resolution images and 3D feature localization

**DOI:** 10.1088/1361-6560/ad131a

**Published:** 2024-01-10

**Authors:** Lennart Volz, Christian Graeff, Marco Durante, Charles-Antoine Collins-Fekete

**Affiliations:** 1 Biophysics, GSI Helmholtz Center for Heavy Ion Research GmbH, Darmstadt, Germany; 2 Department of Electrical Engineering and Information Technology, Technical University of Darmstadt, Darmstadt, Germany; 3 Department of Condensed Matter Physics, Technical University of Darmstadt, Darmstadt, Germany; 4 Department of Medical Physics and Biomedical Engineering, University College London, London, United Kingdom

**Keywords:** particle imaging, particle radiography, proton imaging, ion imaging, focus stacking, spatial resolution, depth detection

## Abstract

*Objective.* We demonstrate a novel focus stacking technique to improve spatial resolution of single-event particle radiography (pRad), and exploit its potential for 3D feature detection. *Approach.* Focus stacking, used typically in optical photography and microscopy, is a technique to combine multiple images with different focal depths into a single super-resolution image. Each pixel in the final image is chosen from the image with the largest gradient at that pixel's position. pRad data can be reconstructed at different depths in the patient based on an estimate of each particle's trajectory (called distance-driven binning; DDB). For a given feature, there is a depth of reconstruction for which the spatial resolution of DDB is maximal. Focus stacking can hence be applied to a series of DDB images reconstructed from a single pRad acquisition for different depths, yielding both a high-resolution projection and information on the features’ radiological depth at the same time. We demonstrate this technique with Geant4 simulated pRads of a water phantom (20 cm thick) with five bone cube inserts at different depths (1 × 1 × 1 cm^3^) and a lung cancer patient. *Main results.* For proton radiography of the cube phantom, focus stacking achieved a median resolution improvement of 136% compared to a state-of-the-art maximum likelihood pRad reconstruction algorithm and a median of 28% compared to DDB where the reconstruction depth was the center of each cube. For the lung patient, resolution was visually improved, without loss in accuracy. The focus stacking method also enabled to estimate the depth of the cubes within few millimeters accuracy, except for one shallow cube, where the depth was underestimated by 2.5 cm. *Significance.* Focus stacking utilizes the inherent 3D information encoded in pRad by the particle's scattering, overcoming current spatial resolution limits. It further opens possibilities for 3D feature localization. Therefore, focus stacking holds great potential for future pRad applications.

## 1. Introduction

Particle imaging is currently being developed as a promising tool for planning CT imaging and online treatment verification in particle therapy, where high precision is a key requirement (Graeff *et al* 2023). The rationale for particle imaging is to use the particle beam both for applying the treatment and acquiring the image, reducing registration and image conversion uncertainties. Measuring the energy loss of the particles after they traversed the patient enables to reconstruct radiography projections of the patients water equivalent thickness (WET), and, when acquiring a tomography, a volumetric map of the relative stopping power (RSP) as required for treatment planning.

Two general approaches to acquire particle imaging have been developed: measuring the integral signal of a large number of particles for image acquisition (denoted integrating mode), or measuring each particle individually on an event-by-event basis (denoted single-event, list-mode or tracking) (Poludniowski *et al* 2015). For single-event particle imaging, a number of tracking detectors is used before and/or after the object, allowing the use of path estimation techniques (Schulte *et al* 2008, Collins-Fekete *et al* 2015, Krah *et al* 2018) to address the multiple Coulomb scattering of the particles inside the patient. Several single-event particle imaging systems are currently in use by different groups (Johnson *et al* 2017, Civinini *et al* 2020, DeJongh *et al* 2020) or currently under development (Alme *et al* 2020, Meyer *et al* 2020). Compared to integrating mode, single-event particle imaging is more dose-efficient and offers overall improved image quality. Single-event particle CT has been experimentally verified to retrieve improved RSP accuracy compared to single-energy x-ray CT and similar RSP accuracy compared to dual-energy x-ray CT (Dedes *et al* 2019, Bär *et al* 2022). The advantage over single-energy x-ray CT is especially notable in the presence of metal implants (Civinini *et al* 2020). The accuracy of single-event particle CT also provides for a high accuracy in range prediction in animal tissues (Volz *et al* 2021).

Nevertheless, while promising, the application of single-event particle CT for direct treatment planning is still limited by relatively long acquisition times in the order of few minutes (Schultze *et al* 2021). Single-event particle radiography (pRad), however, can already be accomplished within few seconds with existing systems. Even sub-second acquisition times may be possible for small fields of view. pRad can be a great asset in the clinical workflow at particle therapy centers, enabling to check for changes in the patient’s anatomy (Sarosiek *et al* 2021), optimizing the conversion of the x-ray planning CT to RSP (Collins-Fekete *et al* 2017), patient alignment (Palaniappan *et al* 2019), and even fluoroscopy for tumor tracking (Han *et al* 2011).

Currently, the achievable image quality of pRad is limited by the particles’ scattering (Volz *et al* 2020). Some of the above listed tasks of pRad, like optimizing the x-ray CT conversion, can and possibly should be performed using the full information on the particles path through the object directly, rather than first reconstructing an image. Still, much of the infrastructure in a clinical environment, like alignment procedures or tumor tracking algorithms, are built for x-ray projection images, and are subject to the quality of the underlying images. In addition, one should not underestimate the value of a visual feedback for the treating physicians, who make decisions based on visual assessment of observed changes. For future clinical translation of pRad, the improvement of image quality is therefore a key objective.

Several analytical and iterative algorithms have been put forward for achieving high quality pRads. Ordonez *et al* have proposed an iterative optimization algorithm to obtain the pRad image from the data (Ordoñez *et al* 2019). As analytical pRad algorithms, the single-event data can, e.g. be binned to radiographs based on the particles measured position at the front tracker (front tracker binning; FTB) or the rear tracker (rear tracker binning; RTB), or any position in between using an estimate of the particles path (distance-driven binning; DDB (Rit *et al* 2013, Gehrke *et al* 2018)). In case of DDB, the spatial resolution for a certain feature is best, when the depth of reconstruction approximately coincides with the depth of the feature (Rit *et al* 2013, Volz *et al* 2020, Khellaf *et al* 2022). To achieve highest image quality therefore requires prior information on the imaged object. Collins-Fekete *et al* (2016) have presented a method to retrieve a high quality pRad image without the need for prior information by estimating the contribution of each particle’s water equivalent path length to each image pixel. While providing high quality images, their method does not retrieve the best quality possible for all features in the object simultaneously (Volz *et al* 2020). In fact, no available analytical pRad reconstruction technique can produce a high spatial resolution for features located at different depths in the object in a single image. This is due to the projection of the scattered particle paths onto a single image plane which causes inevitable loss of information (Volz *et al* 2020).

The purpose of this work was therefore to develop an algorithm which can achieve a single projection image with the highest quality for all features possible from a single pRad acquisition. To achieve this, we relied on focus stacking (FS), a technique common in optical photography and microscopy (Sigdel *et al* 2016). FS was applied to DDB images reconstructed from the pRad data at different depths (DDB stack). This enabled to extract the depth of best image quality for each feature, and then collapse the DDB into a single, high-quality pRad image. This manuscript presents the results achieved with this technique for Monte Carlo simulated pRads of a simple water tank with bone cube inserts and of a lung cancer patient. In addition, the possibility to infer the radiological depth of features in the patient with the FS methodology is presented and its implications on image guided particle therapy discussed.

## 2. Methods

### 2.1. Focus stacking

The basic objective of FS is to combine images acquired at different depths of field into a single image such that the overall spatial resolution is maximized. The most basic algorithm for FS is to construct the final image on a pixel-by-pixel basis (Sigdel *et al* 2016). Suppose a series *f* ^*n*^ of *N* images acquired at different depths of field is available, each with the same dimension *I* × *J*. For each pixel (*i*, *j*) ∈ {*I*, *J*}, the FS image *f* ^*fs*^ contains the pixel’s value from the image *n* ∈ {1,…,*N*} for which the spatial resolution at that pixel location was maximum. Using the value of the Laplacian (denoted as △) of the image as measure of spatial resolution, this can be written as:

\begin{eqnarray*}{f}_{i,j}^{{fs}}=\arg \mathop{\max }\limits_{{f}_{i,j}^{n}}\left(| \bigtriangleup {f}_{i,j}^{n}| \right),\quad n\in \{1,\ldots ,N\}\end{eqnarray*}

**Figure 1.**
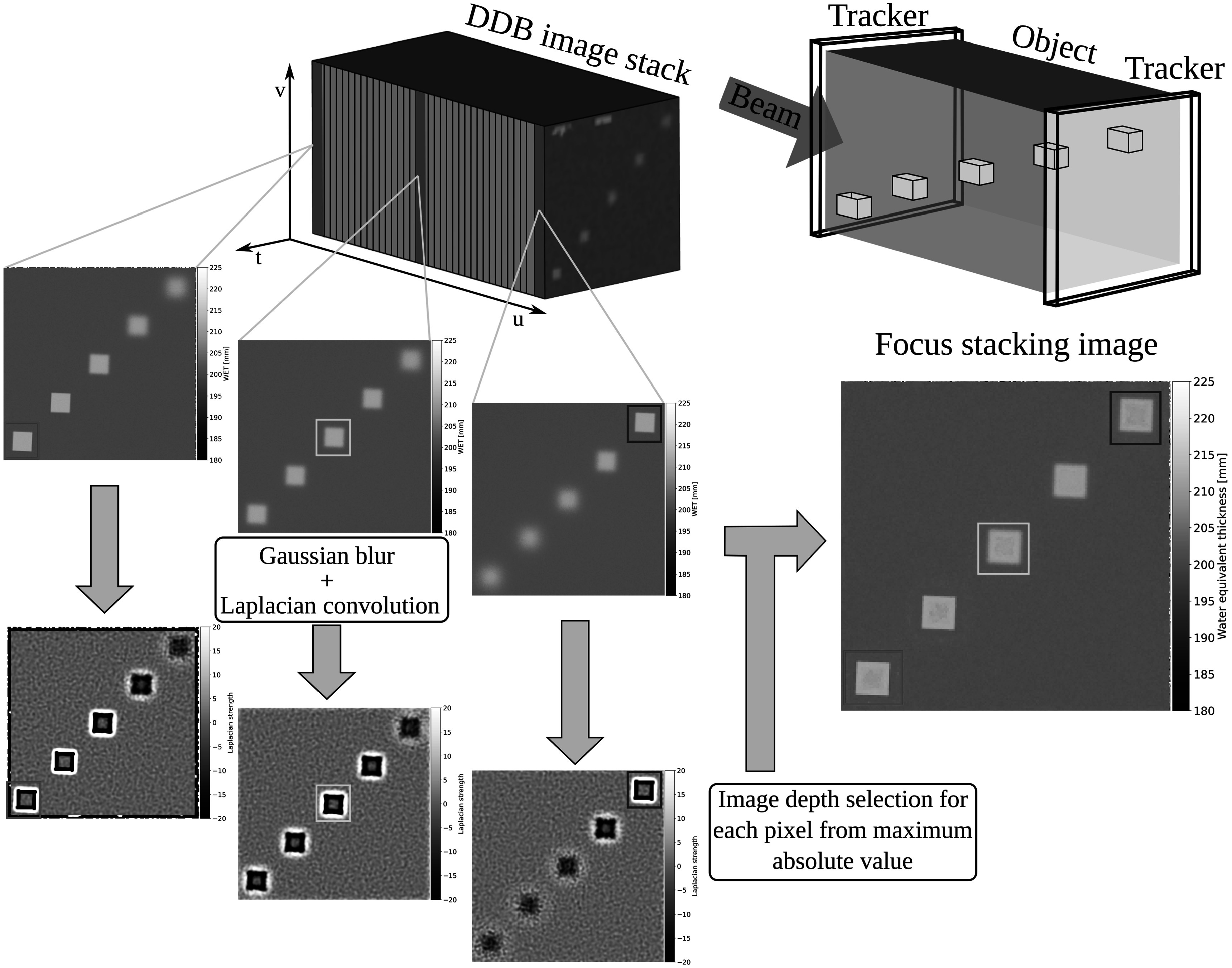
Overview over the workflow for the proposed focus stacking technique illustrated for an example water tank containing 5 cubical inserts at different depths in beam direction (u). First, from the single acquired pRad of the object, DDB images are reconstructed at different depths along the beam direction. For each image in this stack, a Gaussian blur and subsequent convolution with a Laplacian kernel is performed. For each pixel, the image plane with the maximum absolute value for that pixel after the Laplacian convolution is chosen for the final focus stacking image. The process can be schematically followed for the cubes in the red, green and blue squared regions. These are sharpest at the front, the middle and the rear, respectively. These depths are chosen and combined into the final image.

To adapt this technique to single-event particle radiography, we utilize the inherent 3D information contained in the particles path. The particle path estimate enables to bin the particles at different radiological depths in the object according to their estimated lateral displacement at that depth, best known as distance-driven binning (DDB) (Rit *et al* 2013). The spatial resolution of DDB is subject to the uncertainty associated with the most-likely path, as well as the scattering of the particles between the binning depth and the depth of the feature. The spatial resolution of a feature is greatest for binning depths close to the depth of the feature (Rit *et al* 2013, Volz *et al* 2020, Khellaf *et al* 2022). The scattering displacement between the depth of the imaging plane and the feature depth results in a blurring effect otherwise. A stack of DDB projections, reconstructed from a single radiography acquisition at various depths along beam direction, in theory, contains a unique ‘plane of focus’ for each image feature^5^ 
^5^
Compare figure 12 in Volz *et al* (2020), figure 5 in Rit *et al* (2013) and figure 5 in Khellaf *et al* (2022). and, hence, enables to apply a FS technique for spatial resolution improvement. The overall workflow is presented in figure 1.

In this work, we relied on the optimized cubic spline path formalism (Collins-Fekete *et al* 2015) for estimating the particles trajectory through the object. We reconstructed a DDB projection at every 1 mm step, yielding a DDB stack. To determine the plane of focus for each image pixel, we obtained an approximation of the local second order derivative of the image by applying a convolution with a Laplacian kernel. In order to avoid noise oversampling, we applied an image blur in the form of a convolution with a Gaussian kernel prior to applying the Laplacian operator. Here, a kernel size of 5 × 5 was used for the two convolution operations, respectively. The plane of highest spatial resolution was then obtained as the plane where the absolute value of the Laplacian convoluted image was largest. The final pixel value in the FS image was taken the pixel value in the DDB stack at the so-determined focal plane. In terms of equation (1), this means that ${f}_{i,j}^{n}=\mathrm{DDB}{({u}_{n})}_{i,j}$, with DDB(*u*) reflecting the DDB projection binned at depth *u* and *u*_*n*_ representing the *N* discrete depths used to create the DDB stack. To further avoid erroneous estimation of the maximum of the Laplacian value with depth, for each pixel, the Laplacian values as function of depth were smoothened with a Savitzky-Golay filter. The code for the FS technique is openly available at git.gsi.de/lennart.volz/focus-stacking.

### 2.2. Monte Carlo simulation

Monte Carlo simulated particle radiography data was generated with the Geant4 toolkit version 10.7 (Agostinelli *et al* 2003, Allison *et al* 2006, 2016). The QGSP_BIC_HP_EMZ physics list was active for all simulations, as recommended for medical applications by the Geant4 collaboration.

#### 2.2.1. Phantoms

Two setups were considered: a simplified geometry of a 20 × 20 × 20 cm^3^ water tank (G4_WATER) with five cubic bone inserts of 1 cm edge length tilted 2.5° degree around the beam axis. The cubes were staggered laterally, with a horizontal and vertical distance of 20 mm, and longitudinally, being placed at radiological depths of 10 mm, 50 mm, 100 mm, 150 mm and 190 mm. This geometry was used to obtain the spatial resolution of the different reconstruction methods for the five different depths. The bone cubes were made of CB230, at a density of 1.34 g cm^−3^ and RSP of 1.27. The material composition was taken from Watanabe (1999).

The second setup considered a lung cancer patient, procured from the 4D Lung Imaging of NSCLC Patients (4D-Lung) dataset (Hugo *et al* 2017) available in the cancerimagingarchive.net online database (Clark *et al* 2013). The patient dataset is fully described in (Balik *et al* 2013) and (Roman *et al* 2012). For demonstrating the FS technique, one phase of the 4D-CT of the patient was evaluated only, to avoid additional complexity arising from motion artifacts. The CT had a dimension of 512 × 512 × 176, at a pixel size of 0.97 mm and a slice thickness of 2.5 mm. The CT was loaded into the Geant4 simulation as a parameterised volume, using the G4PVParameterized class. To assign materials to each CT voxel in the simulation, the CT was first converted to RSP with a generic Hounsfield Unit lookup table (HLUT). Note, that this did not reflect the correct RSP of each voxel, since the HLUT was not specific to the scanner used for the patient, but this is not expected to influence the validity of conclusions drawn from this simulation. The material of each voxel was defined as either water or air, depending on the RSP, where G4_AIR was used for RSPs up to 0.3, and water for everything else. The water density in each voxel was scaled according to the voxel RSP, the ionization potential was set to 78 eV. This setup enabled to test the radiography reconstruction technique developed in this work on a heterogeneous scenario, close to clinical reality.

#### 2.2.2. Scoring

Ideal phase space scorers were used to measure the track information for every event, storing the relevant quantities in a ROOT (Brun and Rademakers 1997) TTree. These scorers did not have a material budget, and no uncertainty arising from pixel size was assigned to them. Moreover, the particle residual energy was scored directly on the rear tracker. The scorers were directly attached to the surfaces of the water tank for the bone cube phantom. For the lung patient, the scorers sat right at the edges of the parameterized volume generated from the CT. Because of this, there was a ∼10 cm air gap between the scorers and the patient.

The water equivalent thickness traversed by the particle was obtained by integrating over the inverse of the stopping power in water from initial to residual energy. For this, tabulated stopping power values were generated with the same Geant4 simulation setup in advance.

#### 2.2.3. Beam settings

Simulations were done with protons irradiated in a realistic pencil beam spot-scanning field. Each pencil beam had a gaussian beam shape with 3 mm standard deviation and a gaussian angular divergence with 2 mrad standard deviation. The lateral spacing was 3 mm between adjacent spot centers. For the bone cube phantom, the field size was 10 × 10 cm^2^, large enough to contain all cube inserts, and 10^7^ primaries were irradiated per run at an initial energy of 200 MeV. For the patient setup, the field size was set to be 30 × 30 cm^2^, and 3 × 10^7^ primary protons were generated at 230 MeV initial kinetic energy. The particles were generated right in front of the front tracker.

### 2.3. Image reconstruction and evaluation

As a reference for the current state-of-the-art single-event radiography reconstruction techniques, we compared the FS result to individual DDB (Rit *et al* 2013) images at different binning depths, as well as the maximum likelihood particle radiography reconstruction (MLR) technique (Collins-Fekete *et al* 2016). All images were reconstructed with a pixel size of 0.5 × 0.5 mm^2^. Only hits by protons were analyzed during the image reconstruction, assuming the availability of particle identification techniques (Volz *et al* 2018, 2019). In addition, 3*σ* (Schulte *et al* 2005) filters were applied to the angular displacement and WET of the particles.

In order to quantify the image quality, we evaluated the spatial resolution and the inter-pixel noise in homogeneous phantom regions. The spatial resolution was obtained from a slanted edge technique. At each edge of each cube insert of the bone cube phantom, an oversampled edge spread function (ESF) was obtained using the ImageJ image processing software. The four ESFs were averaged and then fitted with an error function. The derivative of the error function yielded the line spread function, the Fourier transform of which produced the modulation transfer function (MTF). As in other works (Seco *et al* 2013, Krah *et al* 2018), we report the spatial frequency at the 10% fall-off of the MTF as a measure of the spatial resolution.

### 2.4. Feature depth detection

The expected unique relationship between the point of highest spatial resolution and the feature depth for a given setup (particle type, initial kinetic energy, detector setup) invites to use the infrastructure for the FS technique presented above also for detecting the radiological depth of a feature. This is motivated by the spatial resolution as function of depth for a given feature as observed for DDB binning: when binning at the feature depth the spatial resolution for the feature will be subject to the uncertainty of the estimate of the particles’ trajectories. At other depths, the displacement of the particles between the feature and the binning depth adds further spatial uncertainty. Since past works (Rit *et al* 2013, Volz *et al* 2020, Khellaf *et al* 2022) have indicated the focal plane to be close to the depth of the feature, the image plane of the highest spatial resolution for a given pixel could be interpreted as the radiological depth of this particular pixel. This allows to compute a projection of the estimated radiological depth, possibly providing insight into the 3D location of different features. For a proof-of-concept, the accuracy of feature depth estimation possible with this technique was assessed based on the bone cube phantom.

## 3. Results

### 3.1. Staggered cubes phantom

**Figure 2.**
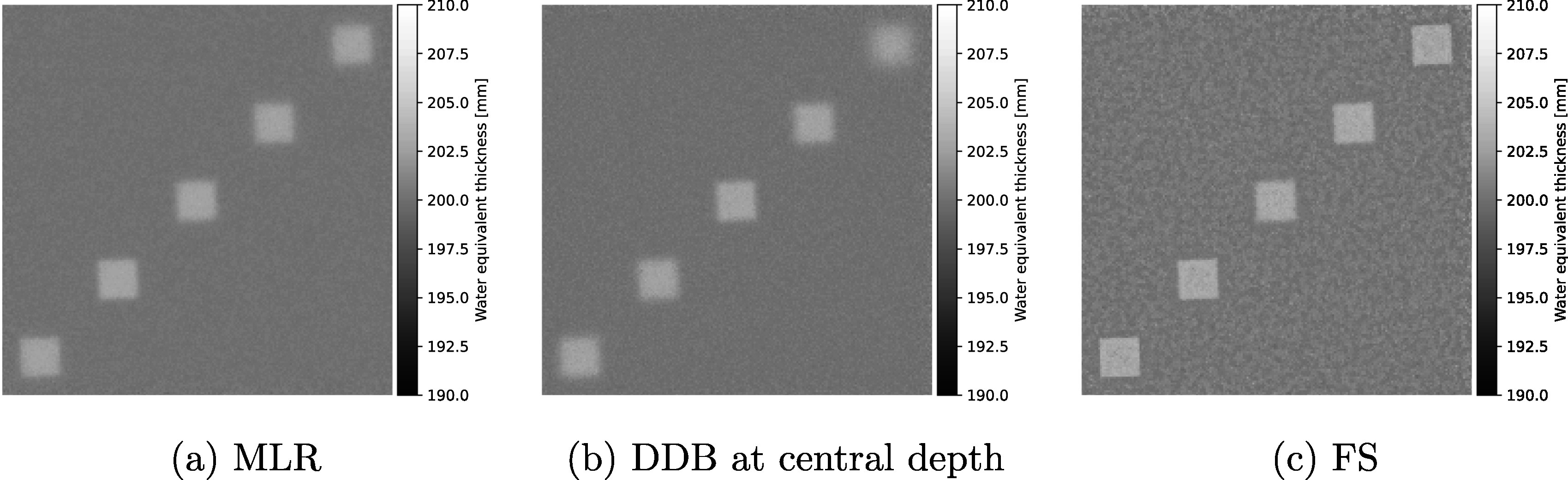
Comparison of different particle radiography reconstruction techniques for proton radiography data of the staggered bone cube phantom. The pRad was acquired with 10^7^ protons at initial energy of 200 MeV.

In figure 2, different proton radiography reconstructions of the bone cube phantom are presented. Figure 2(a) shows the reconstruction with the MLR formalism (Collins-Fekete *et al* 2016), figure 2(b) the DDB image at the central depth in the water tank. Figure 2(c) shows the reconstruction with the FS technique.

**Figure 3.**
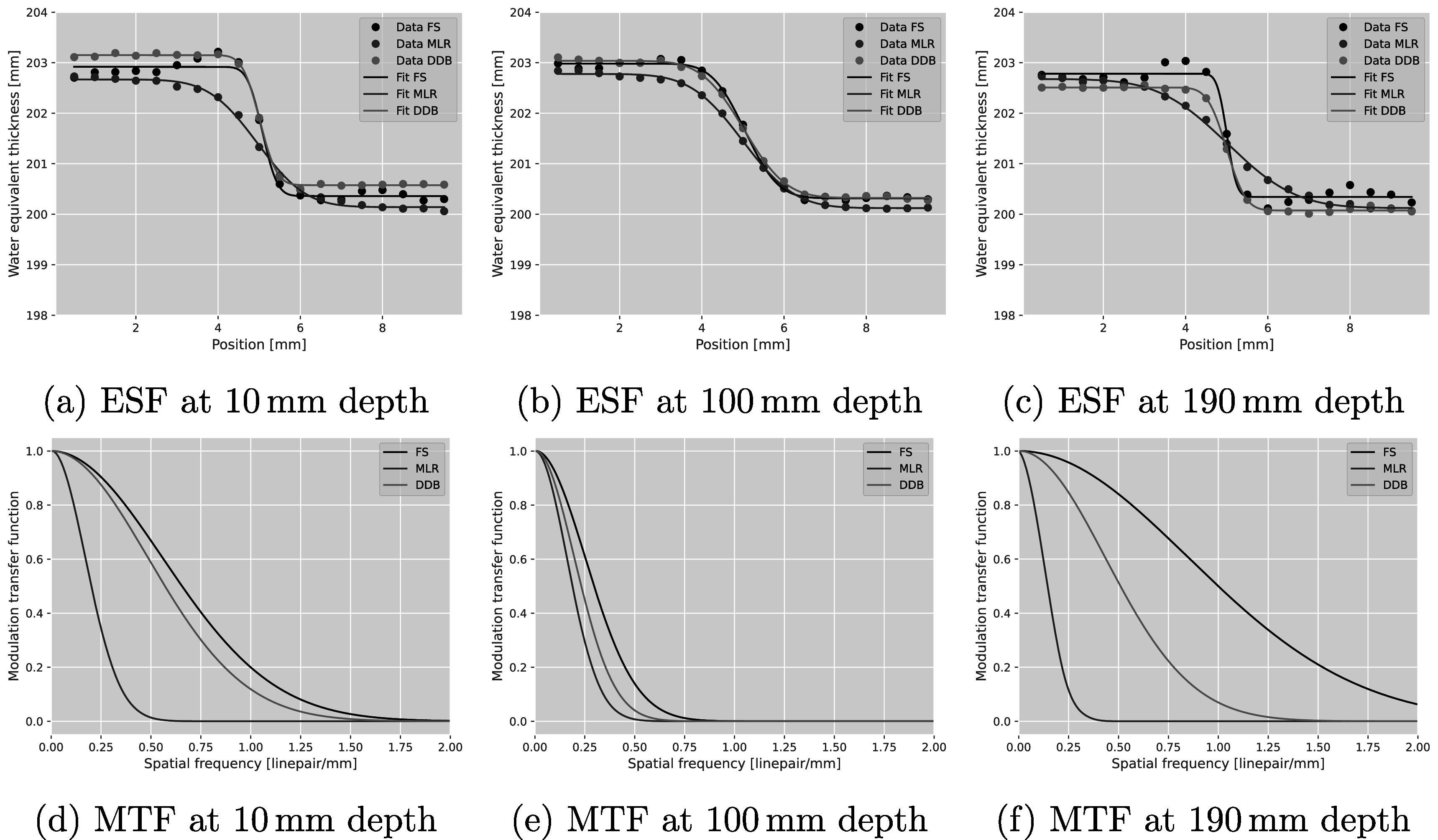
ESFs (top) and the MTFs (bottom) derived from them for a pRad of the bone cube phantom that was acquired with 10^7^ primary protons at 200 MeV initial energy. The pRad was reconstructed using the MLR (red), DDB (green) and the new FS (blue) methods. For DDB, the shown curves correspond to the case where the binning depth was the same as the respective cube’s central depth.

**Figure 4.**
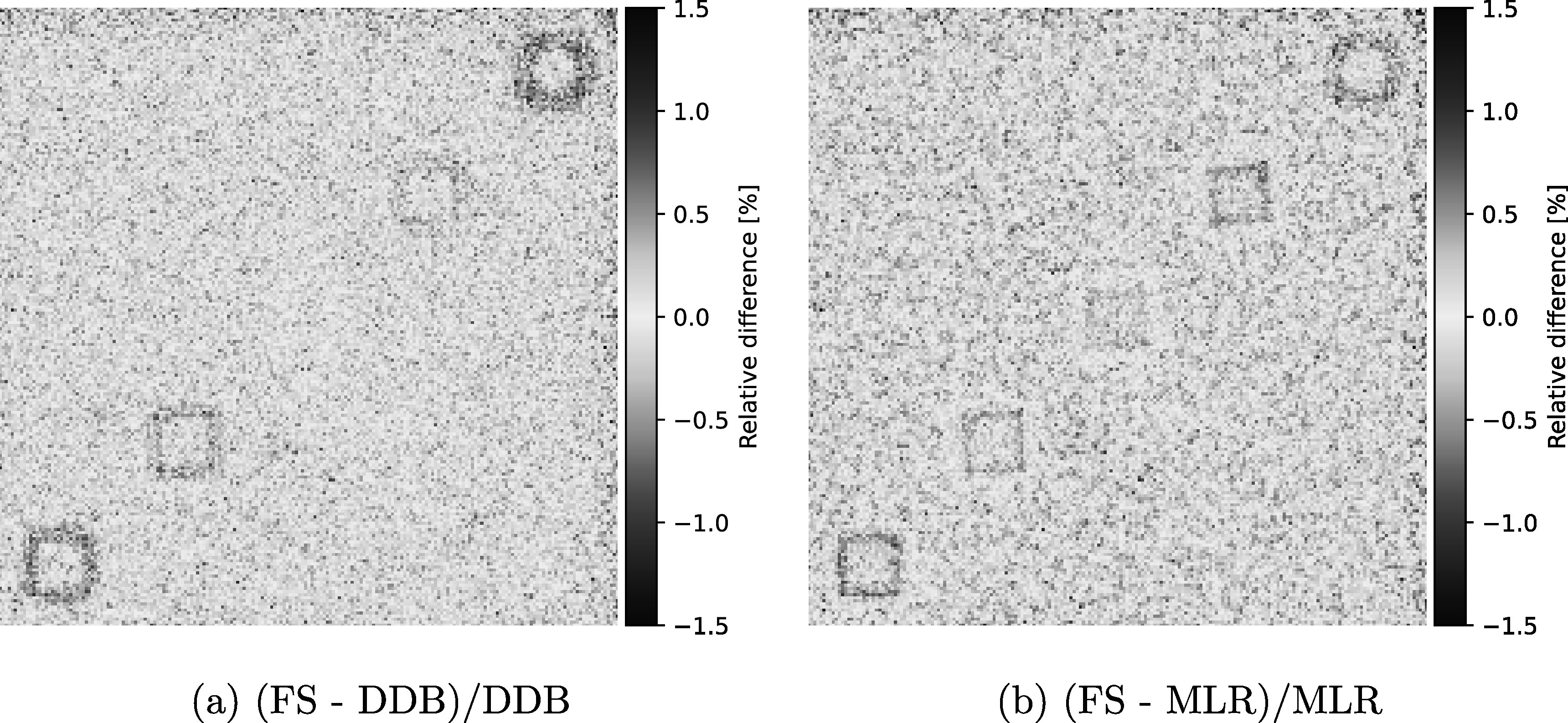
Difference in the WET projections reconstructed with the FS and DDB (with the central depth in the object as binning plane), and the MLR method from a pRad of the cube phantom acquired with 10^7^ primary protons irradiated at 200 MeV initial energy.

Visually, the FS reconstruction presented a clear spatial resolution benefit over both the MLR technique and the central image in the DDB stack. The top-row images in figure 3 show the edge data from the different reconstructions (MLR, DDB at the respective cube depth, and FS) together with the corresponding error function fit. Note the small over-/undershoot in the WET values in the edges of the cubes at 10 mm and 90 mm depth for the FS technique. Similar edge enhancement was observed for the cube at 50 mm depth, albeit less pronounced. The bottom row of the figure shows the MTF curves derived from the error function fits. The MTF10% for each of the five cubes are listed in table 1 the FS technique, as well as the MLR and the respective DDB images at the cube depths. The new FS technique yielded a 7-fold improvement in spatial resolution over the MLR technique for the deepest cube in this setup. The median spatial resolution improvement over the MLR method was 136%; on average, the spatial resolution was improved by 231%. Interestingly, even compared to the DDB images, reconstructed at the depth of the cube object, the spatial resolution achievable with FS was improved. The median spatial resolution improvement achieved with FS over DDB was 28%, while the average spatial resolution improvement was 42%. Figure 4(a) shows the relative percentage difference between the MLR and FS pRad, and respectively figure 4(b) that between DDB at central depth and FS. The differences are limited to the edge regions of the cubes, with the mean difference in a 25 × 25 mm^2^ homogeneous water region being 0.13% compared to the MLR method and 0.01% compared to the DDB image at central depth.

**Table 1.** MTF_10%_ analyzed for a pRad of the bone cube phantom that was acquired with 10^7^ primary protons at an initial kinetic energy of 200 MeV and reconstructed with different radiography reconstruction methods. The reported relative difference values were calculated as $100\times ({{\mathrm{MTF}}}_{10 \% }^{{FS}}/{{\mathrm{MTF}}}_{10 \% }^{{Mod}.}-1)$, where ${Mod}.$ refers to the state-of-the-art image reconstruction reported to the left of the respective value. For DDB, the reported spatial resolution values correspond to a DDB image binned at the same depth as that of the cube.

Cube depth	FS	MLR	Rel. difference	DDB	Rel. difference
10 mm	1.19 lp mm^−1^	0.37 lp mm^−1^	+226%	1.04 lp mm^−1^	+15%
50 mm	0.96 lp mm^−1^	0.41 lp mm^−1^	+136%	0.60 lp mm^−1^	+60%
100 mm	0.54 lp mm^−1^	0.34 lp mm^−1^	+56%	0.42 lp mm^−1^	+28%
150 mm	0.63 lp mm^−1^	0.28 lp mm^−1^	+121%	0.57 lp mm^−1^	+10%
190 mm	1.82 lp mm^−1^	0.26 lp mm^−1^	+613%	0.93 lp mm^−1^	+96%

One drawback of the FS technique in its current form is that the pixel-wise evaluation of the Laplacian is prone to enhancing image noise. Here, the inter-pixel noise was evaluated as the standard deviation of the pixel values in a 25 × 25 mm^2^ region in the homogeneous part of the bone cube phantom. For the MLR it was 0.17 mm, for DDB it was 0.23 mm on average, while it was 0.40 mm for the FS technique. The relationship between the number of primaries and the noise in FS was further explored and is presented in the next section.

#### 3.1.1. Impact of number of primaries

**Figure 5.**
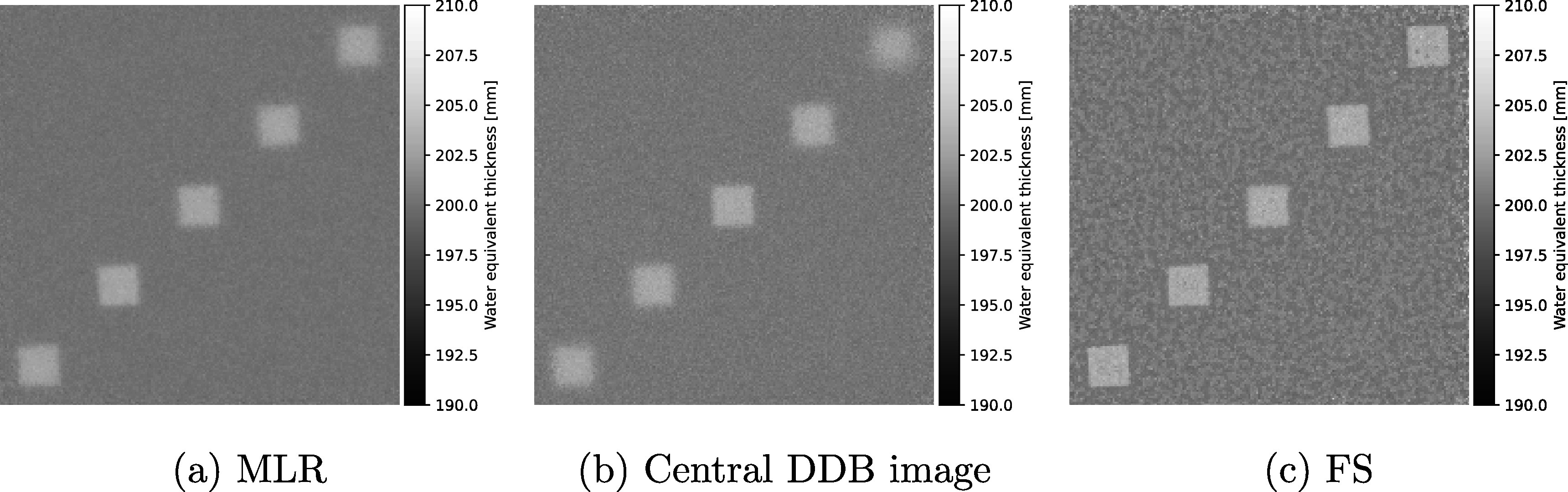
Comparison of MLR (a), central DDB image (b) and FS (c) pRads of the cube phantom for 5 × 10^6^ primary protons. The beam energy was 200 MeV, the irradiation field was 10 × 10 cm^2^.

Figure 5 shows a pRad of the bone cube phantom acquired with 5 × 10^6^ primary protons. The beam initial kinetic energy was 200 MeV.

FS elevated the image noise with reduced statistics compared to the MLR and DDB pRads. The WET standard deviation for FS was 0.55 mm in a 25 × 25 mm^2^ homogenous image region, while for the MLR technique, the same region had a WET standard deviation of 0.23 mm, and for the DDB images, it was 0.33 mm on average. For even less particles (10^6^ primary protons), the focus stacking technique failed to produce an acceptable image, since it resulted in excessive noise (standard deviation 2.1 mm).

### 3.2. Lung patient

**Figure 6.**
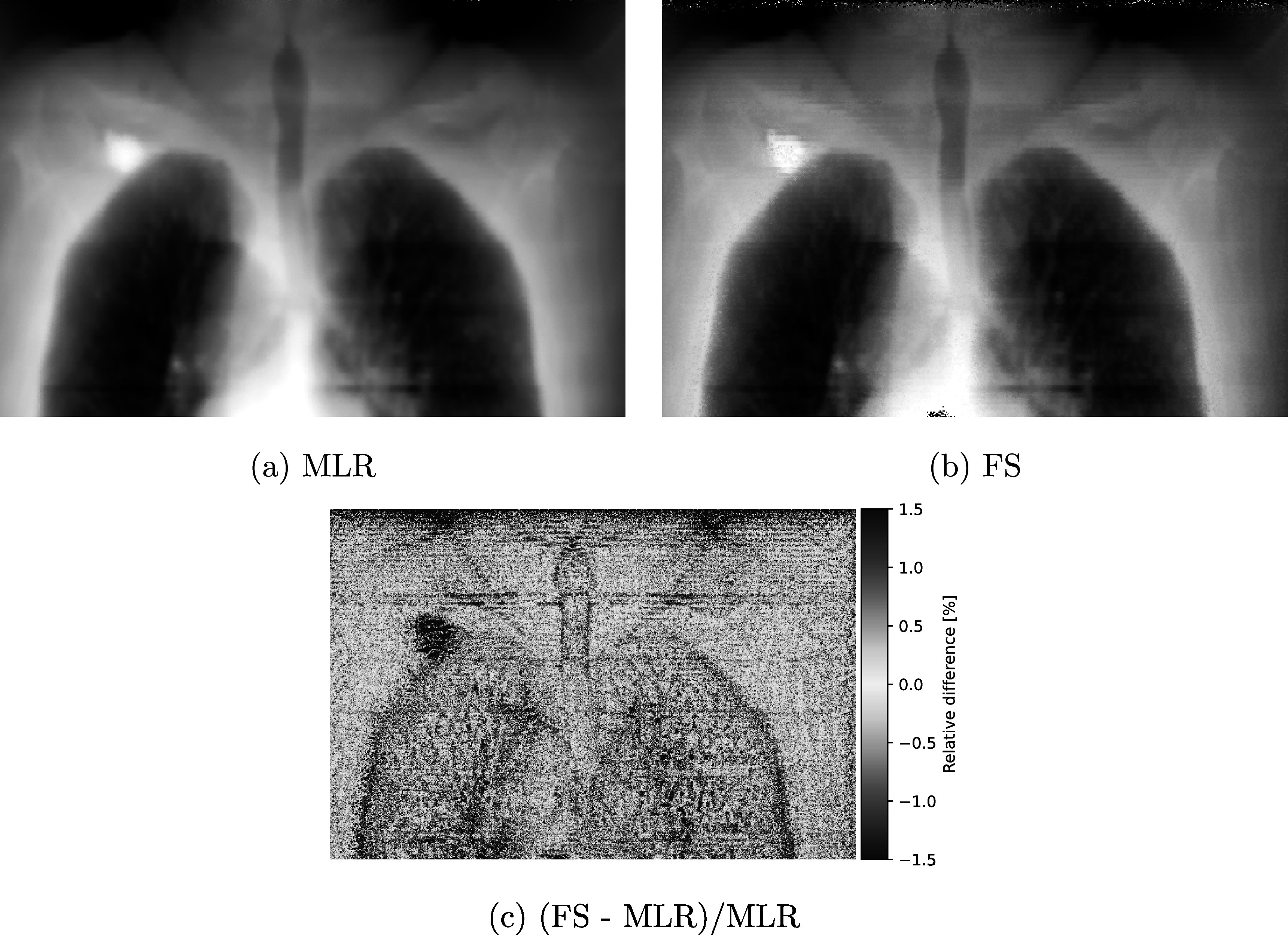
Comparison of the MLR reconstructed pRad of the lung cancer patient and the FS reconstruction. The pRad was acquired with 3 × 10^7^ primary protons at 230 MeV initial energy in a 30 × 3 cm^2^ pencil beam scanning field.

Figure 6 presents the pRads of the lung patient, acquired with 3 × 10^7^ primary protons at 230 MeV kinetic energy, irradiated in a 30 × 30 cm^2^ pencil beam scanning field. The pRads were reconstructed with the MLR (Figure 6(a)) and the FS (figure 6(b)) technique. Due to the lack of a depth of reference for this patient, showing a single DDB image would not be a representative comparison.

Despite the complex anatomy with many overlapping structures, FS also in this case improved the spatial resolution visibly. This can be observed at the small lung structures, as well as the trachea, which are depicted visibly sharper in the FS image. In particular, in the FS image, the voxelized nature of the underlying patient CT can be seen in the final image, indicating that with FS, the spatial resolution is as good as the limitation imposed by the data.

Figure 6(c) shows a relative difference map between the MLR and FS reconstructed images of the lung patient pRad. Differences are largest at edge regions in the patient, while in homogeneous regions differences fluctuate around zero.

### 3.3. Feature depth detection

**Figure 7.**
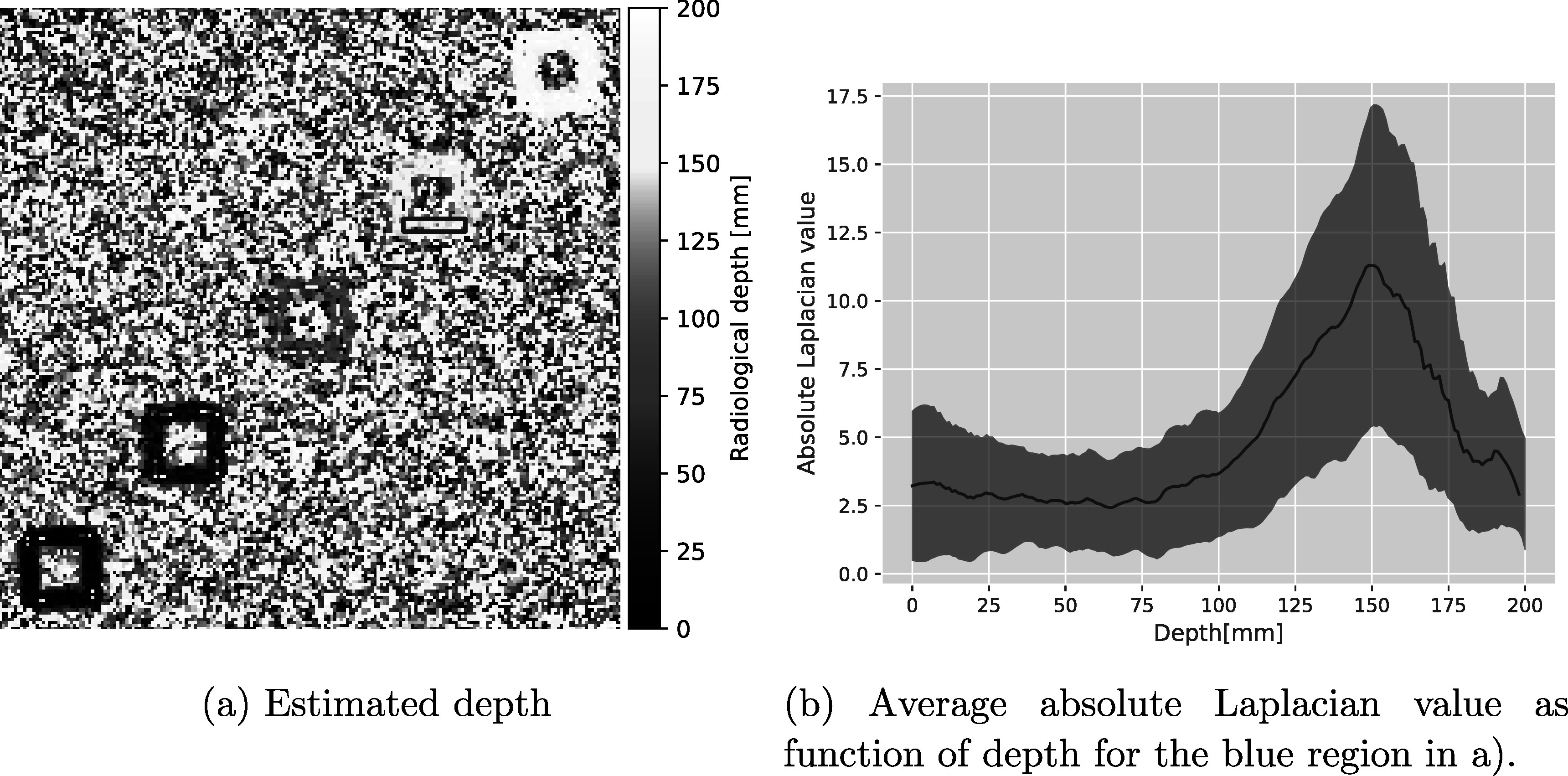
(a) 2D map of the radiological depth where the DDB had the highest spatial resolution for each image pixel evaluated for a pRad of the bone cube phantom that was acquired with 10^7^ primary protons irradiated at an initial energy of 200 MeV through a pencil beam scanning field. The five prominent features correspond to the bone cube edges. From bottom left to top right, the cubes were placed at depths of 10 mm, 50 mm, 100 mm, 150 mm and 190 mm. The blue box indicates an example region used for depth estimation. (b) Shows the average absolute value of the Laplacian as function of depth for the region outlined in blue in (a), i.e. for 80 pixels at the edge of the cube at 150 mm depth. The blue shaded region indicates a one-standard-deviation band around the mean.

In figure 7 the depth of the maximum absolute Laplacian strength is shown for the pRad of the staggered cube phantom, acquired with protons at 200 MeV initial energy, i.e. corresponding to the FS image shown as figure 2(c). It can be seen that the technique differentiates the depth of the cube edges according to their relative depth in the water tank. The edges of the most shallow cube (10 mm depth) on the bottom left hand side are assigned a very shallow reconstruction depth, while for the deepest cube (190 mm depth), the edges are assigned a reconstruction depth in a similar region to their position.

**Figure 8.**
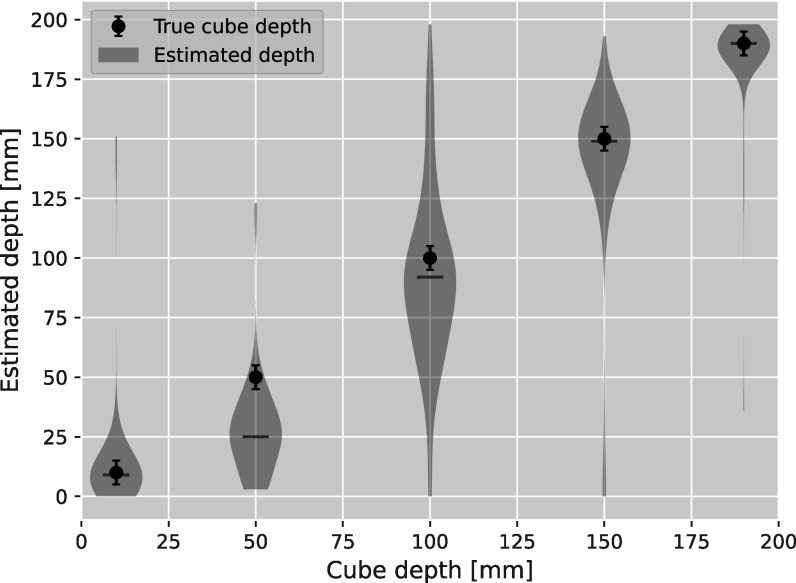
Depth estimated from the maximum absolute value of the Laplacian convoluted DDB images in a 80 pixel area around the cubes’ bottom edges (blue box in figure 7), shown as red violin plots. The central horizontal line indicates the median estimated depth. The black points indicate the center position of the cubes in the simulation, and the errorbars indicate the size of the cube edge.

From a more quantitative analysis of 80 pixels (20 pixels in horizontal, 4 pixels in vertical direction) around the bottom edge of the cubes (indicated as the blue box in figure 7(a)), figure 8 depicts the distribution of the depth estimated for these pixels. For the pRad acquired with 10^7^ protons at initial energy of 200 MeV with pencil beam scanning, the depths of the cubes at 10 mm, 100 mm, 150 mm and 190 mm were estimated to within ≤5 mm to their proximal position in beam direction. The cube at 50 mm was estimated to be located 2.5 cm more shallow than it actually was placed. Note, that this method only yields information for parts of the object that contain a high WET gradient. In homogeneous regions, the absolute of the Laplacian convoluted image values is not expected to yield any information, as can also be seen in the large noise in the reconstructed radiological depth for homogeneous water regions.

## 4. Discussion

In this work, we presented FS as a new tool for spatial resolution improvement and feature depth detection with single-event pRad. Originating from optical microscopy and photography, FS can be applied to single-event particle imaging by utilizing the properties of the scattered particle trajectories through the patient. In this proof-of-concept work we evaluated the potential of this method on simulated pRads of a simple water tank with bone cube inserts, as well as a complex lung cancer patient scenario.

FS yielded greatly improved spatial resolution over contemporary analytical pRad reconstruction algorithms. It achieved an up to 7-fold resolution improvement compared to the MLR method by Collins-Fekete *et al* (2016). This advantage comes at no setback for the patient, and does not pose additional requirements on the detector. Instead, FS fully utilizes the available data from the single-event pRad. The proton paths inherently contain 3D information encoded in their scattering (Volz *et al* 2020). Currently available pRad reconstruction techniques project either an integral over the path into a single projection image (Collins-Fekete *et al* 2016, Gehrke *et al* 2018), or use the path information at a distinct depth to bin the particles in order to form the image (Rit *et al* 2013, Gehrke *et al* 2018). The former presents an inevitable loss of information, while the latter requires prior knowledge on the feature location and cannot yield a single high quality image for features located at different depths.

The FS technique presents the first analytical method that uses the 3D particle path information to obtain a single image with the best spatial resolution for features at different depths. Even for the complex case of the lung cancer patient with many overlapping structures, the spatial resolution with FS was visibly improved compared to the MLR pRad, which currently is the only other analytical method that produces a high quality pRad without the need for prior information. Overlapping structures are also not expected to influence the technique too much, since the pixel-wise evaluation of the Laplacian convoluted DDB images will yield the highest gradient for each individual pixel. This results in the sharpest representation for each individual feature possible from the data, regardless whether features overlap or not.

Interestingly, the FS technique presents an advantage even compared to DDB images where the binning depth was the same as the objects depth. This is counter-intuitive, seeing how the FS image is constructed from the DDB images themselves. First, the cubes being 10 mm thick introduces slight ambiguity in the comparison to the DDB projection at the cube’s central depth. This may affect the resolution for the most distal cube, where the resolution drops quickly with increasing distance from the rear tracker. More importantly, the slight edge overshoot was observed for the first and last cube in the FS radiography in figure 3. The over-/undershoot close to the edges is likely related to image noise. Over-/underestimation in the pixels at the cube edge can produce a larger Laplacian value, such that among several images with similar resolution, those are selected where noise enhances the Laplacian value. While this has only a minor effect on WET accuracy, it presents a bias to the comparison with DDB, as it affects the error function fit.

For the cube at 50 mm depth, the median focal depth was found to be at 25 mm depth (see figure 8). For this cube, also a comparatively large improvement in spatial resolution (+60%) was observed in comparison to the DDB radiography at the cubes depth. At the same time the edge overshoot was not as pronounced as for the first cube, where the spatial resolution improvement was smaller (15%). A similar underestimation of the depth of highest focus compared to the feature depth has been discussed in Volz *et al* (2020), where the focal plane was estimated to sit ∼10 mm before the object. The underestimation of 25 mm observed here is larger than what was predicted in that work, and likely related to the chosen settings for the Savitzky-Golay filter. Further investigation is needed to fully model and understand this, especially, when the goal is to use FS for feature depth detection.

For the most shallow and two deepest cubes, the depth could be estimated within the ambiguity given by the cube edge length. This has interesting potential for the use of pRad for pre-treatment verification. It could potentially be used to estimate the depth of fiducial markers and with that for 3D tumor location. The current systematic shift of the depth estimation for shallow objects would mean that this would likely work best for deeper targets. It would likely not require a large field of view and possibly few pencil beam probes may suffice, keeping the irradiated dose low and enabling very fast acquisition. With contemporary single-event pRad systems that have data acquisition rates in the order of 1 MHz, 10^5^ particles can be acquired in ∼0.1 s or less (Johnson 2017). This corresponds to the same approximate fluence as covering a single cube in the bone cube phantom shown in this work, i.e. can be assumed sufficient for depth inference. Especially for fiducial markers, which are typically just few millimeters in size, such an irradiation field would be enough for depth estimation. This could open the door for real-time tumor tracking with pRad at very low doses, although more work is needed to demonstrate this in an experimental setting.

Even if the depth estimation proves to be not accurate enough for 3D feature location, the method could still help to identify whether observed anatomical changes occurred proximal to the tumor, i.e. would influence the treatment, or distal, i.e. can safely be ignored. For this, the accuracy of the method is not as relevant, since only the relative depths matter. It also has to be noted that this method depends on the gradient of the object. The larger the gradient, the clearer the maximum in the Laplacian convoluted images as function of depth. In mostly homogeneous soft tissue regions with similar RSP, there is little to no information and no depth inference is possible. The bone cubes chosen in this work represent a comparatively small difference of only 2.7 mm compared to the surrounding water, i.e. a somewhat realistic case compared to the clinical reality in patients. This work therefore can be seen as a first proof-of-concept indicating promising potential for further investigation.

Overall, although the particles’ scattering is quoted often as one of the key issues with particle imaging, the inherent 3D information encoded into the scattering presents itself as a key feature that can be exploited for additional inference on the patients anatomy. In the future, FS and pRad based depth inference may open new ventures in the field of particle imaging.

One drawback of the FS technique in the form it is used in this work, is that it enhances noise due to the evaluation of the Laplacian strength on a pixel-by-pixel basis. The second order derivative is sensitive to image noise, and other methods like the Sobel operator (approximates the first order derivative) would be more robust. However, the Sobel operator provides the gradient only in one of the two cardinal image directions at a time, such that it is hard to determine the plane of global spatial resolution optimum. Different techniques exist in the field of microscopy, that, instead of relying on a pixel-by-pixel evaluation of image gradients, use image regions to determine the best plane of focus for each feature (Sigdel *et al* 2016). Such techniques could also be interesting in the context of FS applied to pRad and should be investigated in future works. Here, we chose the pixel-by-pixel evaluation of the Laplacian strength due to its simple implementation and the already promising results achieved with it.

Still, the depth of highest absolute Laplacian value is subject to noise fluctuations. In order to improve the FS technique, we smoothened the absolute value of the Laplacian convoluted images as a function of depth. This yielded more stable image quality, and also improved the depth inference. Still, the kernel sizes for the two convolution operations (Gaussian and Laplacian), as well as the parameteres of the smoothening function were chosen based on an empirical investigation of different settings. These likely need fine tuning for the specific setup at hand, which is not ideal for a streamlined imaging workflow. Further development is therefore needed in order to maximize the potential achievable with FS.

## 5. Conclusion

Focus stacking opens new possibilities for particle imaging. It enables to fully utilize the inherent 3D information encoded in single-event pRad data through the scattered particle paths. Not only does Focus stacking provide a high resolution for all features in the object at the same time, it also presents an interesting route toward 3D feature detection with particle imaging. This can aid future pRad guided particle therapy, as it opens the possibility to infer whether an observed change occurred before the target, i.e. would affect the treatment, or not. The improved spatial resolution offered by focus stacking pRad will aid physicians in assessing the patient anatomy and might benefit image registration techniques. At the same time, focus stacking adds only little computational cost. This makes focus stacking a promising tool for future particle imaging applications.

## Data Availability

All data that support the findings of this study are included within the article (and any supplementary information files).
